# Mechanobiology of the tumor microenvironment: a review of therapeutic interactions and in vitro elasticity measurement techniques

**DOI:** 10.1186/s12929-026-01270-x

**Published:** 2026-06-22

**Authors:** Ting-Wei Chen, Shu-Han Yu, Shao-Lun Lu, Chih-Hung Yeh, Pai-Chi Li

**Affiliations:** 1 Graduate Institute of Biomedical Electronics and Bioinformatics, National Taiwan University, Taipei, Taiwan; 2 Institute of Biotechnology, National Taiwan University, Taipei, Taiwan; 3 Graduate Institute of Oncology, National Taiwan University College of Medicine, Taipei, Taiwan; 4 Department of Radiation Oncology, National Taiwan University Cancer Center, Taipei, Taiwan; 5 Department of Electrical Engineering, National Taiwan University, Taipei, 106319 Taiwan

**Keywords:** 3D cell culture, Cancer therapy, ECM stiffness, Elasticity imaging, Shear-wave elastography, Tumor microenvironment

## Abstract

The stiffness of the extracellular matrix (ECM) regulates cellular behavior, influencing tumor progression and therapeutic resistance. In cancer, aberrant ECM stiffening can reduce immune cell infiltration and treatment efficacy, which can in turn alter the tumor microenvironment. Here, we review the role of ECM stiffness in cancer biology and its relevance to matrix-targeted therapies and biomaterial design. We discuss three-dimensional (3D) in vitro models that mimic native tissues and the bidirectional interactions between ECM mechanics and therapeutic interventions. A comparative analysis of measurement modalities is presented for characterizing complex 3D environments, including shear-wave-based techniques such as optical and ultrasound elastography and non-shear-wave-based approaches such as atomic force microscopy and rheology. Future directions include developing matrix-modulating therapies, integrating elasticity sensors into microfluidic devices for higher throughputs and physiological relevance, and applying machine learning to interpret heterogeneous mechanical properties. Collectively, these engineering and biological advances highlight ECM stiffness as a tractable target and open translational opportunities for predictive modeling, diagnostic platforms, and matrix-directed therapies to improve cancer treatment.

## Introduction

The extracellular matrix (ECM) is a dynamic regulator of cellular functions, influencing proliferation, migration, and differentiation through biochemical and mechanical cues. Its elasticity, determined by a network of proteins and biomolecules, is critical for physiological and pathological processes. In cancer, increased ECM stiffness alters cellular dynamics to promote tumor progression, immune evasion, and therapeutic resistance, whereas softer ECM environments can suppress proliferation and survival, underscoring the delicate balance between matrix elasticity and cellular behavior [1, 2].

To investigate these processes, tunable biomaterials have been developed that allow precise control of mechanical properties by adjusting parameters such as polymer concentration and crosslinking density. These systems can replicate physiological stiffness across two-dimensional (2D), three-dimensional (3D), and intermediate 2.5D culture formats, which more faithfully model the tumor microenvironment (TME). This review further examines how ECM stiffening can impair the efficacy of chemotherapy, radiotherapy (RT), and immunotherapy, and how these therapeutic interventions remodel matrix stiffness.

A range of methodologies for assessing tissue elasticity is also reviewed, including bulk- and guided-wave approaches such as magnetic resonance elastography (MRE), optical and ultrasound-based shear wave imaging, and non-shear-wave methods including atomic force microscopy (AFM), rheology, and Brillouin microscopy, considering their respective spatial scales and applications. Future directions include the miniaturization of measurement systems for high-throughput integration and the application of machine learning (ML) for data interpretation. An improved understanding of these biomaterials and measurement technologies may inform the development of predictive preclinical models and matrix-targeted therapies.

## Role of ECM elasticity in mechanotransduction and cellular fate

The ECM is a dynamic network that provides structural integrity and biochemical regulation of the tissue homeostasis. Its mechanical properties, such as elasticity, vary across tissue types and affect cellular function via biomechanical and biochemical interactions. The associated mechanical forces can be categorized based on their orientation into (1) parallel shear stresses, such as blood flow-induced forces on the vascular endothelium, and (2) perpendicular compressive or tensile forces experienced by load-bearing structures such as bones. Even tissues considered mechanically quiescent, such as the brain and mammary glands, experience nanoscale forces transmitted through cell–cell and cell–ECM interactions, indicating that mechanotransduction is a widespread regulatory mechanism.

### ECM elasticity as a key regulator of structural and biochemical cellular functions

**Fig. 1:**
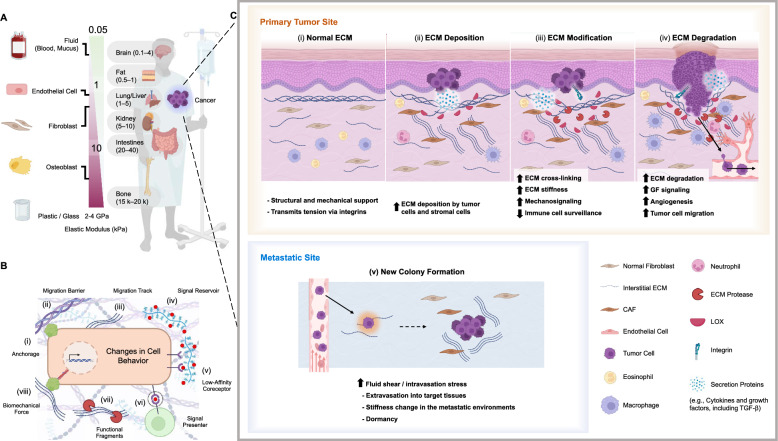
Extracellular matrix (ECM) elasticity and its influence on cellular behavior. **A** Distinct elastic moduli of various tissues in the body. **B** Schematic representation of the multifaceted roles of the ECM: (i) anchorage, (ii) migration barrier, (iii) migration track, (iv) signal reservoir, (v) low-affinity coreceptor, (vi) presentation of signals to cells, (vii) release of functional fragments, and (viii) transmission of biomechanical forces. Collectively, these properties and components of the ECM modulate various aspects of cellular behavior, including adhesion, migration, proliferation, and differentiation. **C** ECM elasticity and tumor progression. (i) In normal tissues, fibroblasts sustain ECM homeostasis and immune cells patrol the compliant stroma. (ii) During tumor progression, cancer-associated fibroblasts deposit collagen and lysyl oxidase (LOX) cross-links fibrils, increasing matrix density and stiffness. (iii) Tumors upregulate integrins and contractility, align fibers, and proteases such as matrix metalloproteinases to open migratory tracks. (iv) Stiff remodeled ECM promotes invasion, basement membrane breach, intravasation, and metastasis. Figure adapted from [7–9]. *GF* growth factor, *CAF* cancer-associated fibroblasts

Elasticity, the ability of a material to undergo nonpermanent deformation, is a mechanical property of the ECM involved in tissue organization, cell adhesion, migration, and signaling. ECM elasticity varies widely across tissue types and directly influences cell phenotypes in a tissue-specific manner. The elastic moduli of soft tissues such as the brain and blood are low (0.05–1 kPa), those of intermediate-stiffness tissues such as skin and muscle range from 1 to 10 kPa, and those of bone reach the order of gigapascals [3, 4] (Fig. 1A). These distinct mechanical microenvironments modulate cellular responses in vivo by altering cytoskeletal tension, gene expression, and cell differentiation [5]. Matrix elasticity strongly regulates mesenchymal stem cell lineage specification; soft matrices promote neurogenic differentiation, intermediate stiffness favors myogenesis, and rigid matrices induce osteogenesis. Although early differentiation remains reversible, prolonged culture results in stable elasticity-dependent commitment. This process is mediated by cytoskeletal mechanotransduction, as the inhibition of nonmuscle myosin II abolishes stiffness-directed lineage specification [6].

In addition to defining tissue elasticity, the ECM performs multiple interconnected biological functions that together regulate tissue organization and cellular behavior [7] (Fig. 1B). As (i) an anchorage, the ECM provides structural support and mechanical stability, transmitting tension through integrins to the cytoskeleton and nucleus to modulate the gene expression and cellular architecture. Acting as both (ii) a migration barrier and (iii) a migration track, their composition and stiffness determine whether they restrict or guide the cell motility. Notably, restoring normal ECM tension can revert malignant cells to a less aggressive phenotype, underscoring its importance in tissue homeostasis. Moreover, as (iv) a signal reservoir and (v) a low-affinity coreceptor, the ECM binds and presents growth factors that regulate the signaling pathways involved in tissue development, regeneration, and disease progression.

In addition to its biochemical signaling roles, the ECM provides dynamic mechanical cues for cells. (vi) By presenting signals to cells, it activates integrin-mediated mechanotransduction cascades, including the MAPK–SMAD and JAK–STAT pathways, thereby influencing cell proliferation, differentiation, and immune responses. Continuous proteolytic remodeling generates (vii) bioactive fragments that act as secondary mediators of intracellular signaling, tissue remodeling, and fibrosis progression. Finally, by (viii) transmitting biomechanical forces, the ECM conveys tension from integrins through the cytoskeleton to the nuclei. Consistent with the tensegrity model, these mechanical forces induce nuclear deformation, chromatin reorganization, and transcriptional changes that drive stem cell differentiation, fibrosis, and tumor evolution. Collectively, the properties and components of the ECM integrate its biochemical and biomechanical functions into a dynamic regulatory network that orchestrates cell adhesion, migration, proliferation, and differentiation, highlighting its central role in mechanosensitive regulation and potential as a therapeutic target in fibrosis and cancer.

## ECM elasticity in the tumor microenvironment

Aberrant ECM remodeling is implicated in diseases such as fibrosis and cancer, where excessive stiffness disrupts normal tissue homeostasis and contributes to poor outcomes. While increased elasticity promotes tumorigenesis, it also impairs drug delivery and immune cell infiltration, complicating therapeutic approaches [10–12]. Research aimed at modulating ECM properties to improve treatment efficacy has highlighted the therapeutic potential of targeting ECM mechanics in regenerative medicine and oncology [13, 14]. Understanding ECM elasticity not only advances fundamental biology but also informs the development of clinical strategies.

### Effects on cancer progression and therapeutic resistance

The TME consists of malignant cells, immune cells, endothelial cells, fibroblasts, adipocytes, and the ECM, which together form a dynamic ecosystem that influences cancer progression. As a structural and biochemical regulator, the ECM provides support for cellular survival, differentiation, and function via its constituents such as polysaccharides, proteoglycans, and fibrous proteins including collagen, elastin, fibronectin, and laminin. These components influence cell morphology, adhesion, and function, thereby affecting tumor growth and metastasis. Increased ECM stiffness is a characteristic of the TME that alters cellular signaling and accelerates tumor progression. Cancer cells actively remodel the ECM by enhancing collagen cross-linking and glycosaminoglycan deposition, generating a mechanically reinforced microenvironment that sustains proliferation, invasion, and metastatic potential.

In normal tissues, fibroblasts maintain ECM homeostasis, whereas immune cells such as eosinophils, macrophages, and neutrophils patrol the interstitial space to preserve tissue integrity (Fig. 1C(ii)). In contrast, solid tumors frequently develop a desmoplastic stroma characterized by excessive collagen deposition and cross-linking, accumulation of glycosaminoglycans such as hyaluronic acid and heparan sulfate, and a marked increase in tissue stiffness (Fig. 1C(ii)). This pathological stiffening, detectable by MRE and ultrasound elastography, contributes to aberrant cell–matrix interactions and tumor progression.

Beyond its structural effects, ECM stiffening alters the biochemical and mechanotransductive landscapes of the TME. Cancer-associated fibroblasts (CAFs) drive this process by secreting lysyl oxidase (LOX), which promotes collagen cross-linking and exerts contractile forces that further increase matrix rigidity. The resulting mechanical stress activates epithelial–mesenchymal transition (EMT) through the integrin–FAK–paxillin–YAP/TAZ and Rho GTPase pathways, enhancing tumor invasion and oncogenic transcriptional programs [3, 15, 16] (Fig. 1C(iii)). CAFs also remodel the ECM by depositing matrix components and releasing proteases such as matrix metalloproteinases (MMPs), which degrade existing ECM structures. This dynamic remodeling disrupts tensional homeostasis, facilitating tumor expansion, angiogenesis, and metastasis while limiting immune cell infiltration and creating an immunosuppressive microenvironment that enables immune evasion [13] (Fig. 1C(iv)).

### The effect of mechanical changes in cancer metastasis

Mechanical changes continue to influence cancer progression after tumor cells escape the primary TME. During hematogenous dissemination, circulating tumor cells (CTCs) are exposed to fluid shear stress in the bloodstream, where forces ranging from approximately 0.5–4 dyne/cm^2^ in veins to 4–30 dyne/cm^2^ in arteries can induce membrane damage, cytoskeletal disruption, and apoptosis [17–19]. Intravital imaging studies have further demonstrated that many tumor cells are mechanically fragmented within the microvasculature, contributing to the extremely low metastatic efficiency of CTCs [20, 21]. However, a subset of CTCs can activate adaptive mechanotransduction pathways that support survival and dissemination under shear stress [22] (Fig. 1C(v)).

Following extravasation, disseminated tumor cells must adapt to the biomechanical properties of distant metastatic environments, which vary substantially across organs [23–25], as described in Fig. 1A. In addition to stiffness differences, organ-specific ECM composition provides distinct biochemical and mechanical cues that regulate tumor cell dormancy, proliferation, and therapy resistance [24–26]. Tumor cells that fail to appropriately adapt to these mechanical environments may remain in a dormant state for prolonged periods before metastatic reactivation [26] (Fig. 1C(vi)). These biomechanical changes have important implications for matrix-targeted therapies. Although ECM-softening strategies may improve vascular perfusion and drug delivery, excessive matrix degradation may unintentionally facilitate cancer cell invasion and intravasation by exposing aligned collagen fibers that serve as migration tracks toward blood vessels [8, 24, 27]. Therefore, matrix-targeted therapies should aim to achieve controlled biomechanical remodeling while minimizing pro-metastatic effects.

### Targeting ECM elasticity as a therapeutic strategy

The relationship between ECM stiffness and tumor progression makes targeting ECM elasticity a therapeutic approach. Several strategies have been explored to modulate ECM-driven tumor dynamics. Inhibiting ECM cross-linking enzymes such as LOX inhibitors [28] reduces collagen cross-linking, thereby decreasing ECM stiffness and tumor progression. Disruption of mechanotransduction pathways by targeting integrin signaling, particularly by administering FAK and YAP/TAZ inhibitors, can mitigate ECM-mediated tumor-promoting effects and increase sensitivity to conventional therapies [29]. Enzymatic degradation of ECM components using agents such as hyaluronidase and collagenase has been investigated to decrease ECM density and improve drug penetration in desmoplastic tumors [10–12]. Additionally, biophysical modulation strategies using biomaterials and force-modulating therapies aim to restore normal ECM mechanics and reprogram the tumor-associated stroma into a less aggressive state. Future research should focus on integrating ECM-targeting strategies with immunotherapy and conventional treatments to optimize cancer management and improve patient outcomes [30].

## In vitro ECM models: materials and dimensionality

Most adherent cells cannot grow in suspension, and culturing cells on ECM in vitro provides a stable substrate that promotes cell attachment and proliferation. Cells respond to matrix components that resemble the in vivo microenvironment, and ECM-derived materials are commonly included in cell culture systems. The ability of ECM molecules to regulate cellular behavior in vitro suggests their involvement in tissue development and homeostasis by influencing processes such as adhesion, growth, morphology, migration, differentiation, neuronal outgrowth, and cellular lifespan.

Biomaterial-based methods have been developed to control matrix stiffness, allowing systematic investigations of mechanotransduction and its effects on cellular behaviors. Commonly used synthetic and natural substrates include polydimethylsiloxane (PDMS), polymethyl methacrylate, polyurethane acrylate, polyacrylamide (PAA) hydrogels, polyethylene glycol (PEG), OrmoComp®, styrenated gelatin, and collagen I. By adjusting parameters such as polymer concentration, cross-linking density, and molecular weight, these materials can be engineered to replicate the mechanical properties of native tissues for studies on cell adhesion, migration, and differentiation (Table 1).

**Table 1:** Summary of biomaterial-based in vitro extracellular matrix (ECM) models

Dimensionality and feature	Methods	Materials	Mimicking	Elasticity (Young’s modulus)	Biological applications	
					Surface treatment	Cell types
2D Flat surface	‒	Silicone rubber (PDMS)	‒	‒	‒	Heart fibroblasts, liver parenchymal cells, pigmented retina cells [31]
	Soft lithography, direct e-beam writing, microcontact printing	PDMS, PMMA	Heart	≈15 kPa	Fibronectin	Human foreskin fibroblasts, rat cardiomyocytes, cardiac fibroblasts [32]
	Replica molding with embedded microbeads	PDMS	Heart	≈16–38 kPa	Fibronectin	Rat cardiac fibroblasts [33]
	‒	PAA	‒	≈5–8.5 kPa	Collagen I	*Dictyostelium discoideum* [34]
2.5D Topography surface	Replica molding	PDMS	Artery	2.5 MPa, 1.6–2.7 nN/μm depending on diameter and height of the posts	Fibronectin, collagen IV	Bovine pulmonary artery smooth-muscle cells, NIH3T3 mouse fibroblasts, bovine pulmonary artery endothelial cells, Madin–Darby canine kidney epithelial cells [35, 36]
	Replica molding	PAA	‒	‒	Poly-L-lysine	Spinal commissural neurons [37]
	Replica molding	PDMS	‒	Spring constant, *k* = 32 nN/μm	Fibronectin	NIH3T3 fibroblasts [38]
3D Matrix scaffold	Two-photon direct laser writing	OrmoComp®	Heart structure	800 MPa	Fibronectin	Primary chicken cardiomyocytes [39]
	Microfabrication and soft lithography	PDMS	Bone	‒	‒	Connective-tissue progenitor cells [40]
	Two-photon laser scanning photolithography	PEG	Skin	‒	Arginine-glycine-aspartic acid	Human dermal fibroblasts [41]
Gradient matrix scaffold	Photolithography	Styrenated gelatin	‒	10–400 kPa	‒	3T3-Swiss albino fibroblasts [42]
	Compressing of wedge-shaped collagen matrix	Collagen I	Skin	1000–2300 kPa	Collagen I	Human dermal fibroblasts [43]
Microfluidic channel microchannel	Soft lithography	PDMS	Vascular networks, tissue barrier	‒ (Variable)	Fibronectin	Lung epithelial cells, endothelial cells, cancer cells [44–46]

*2D* Two-dimensional, *2.5D* intermediate, *3D* three-dimensional, *PDMS* polydimethylsiloxane, *PMMA* polymethyl methacrylate, *PAA* polyacrylamide, *PEG* polyethylene glycol

### Dimensionality and scale of in vitro cell culture models

The dimensionality and scale of in vitro models are important for recreating the native ECM properties. Based on the geometric relationship between the substrates and embedded cells, culture systems can be categorized into 2D, 3D, and intermediate (2.5D) approaches (Table 1). In 2D cell-based assays, cells are cultured as a monolayer on a flat, rigid substrate, which is sometimes coated with fibronectin or collagen I [47], exposing all cells to a homogeneous distribution of nutrients and growth factors in the medium. Despite being a widely used in vitro method for drug screening, 2D models fail to replicate the geometric complexity of in vivo conditions and may yield data that do not predict in vivo responses [48–50]. Cells cultured in 2D environments exhibit distinct differences in morphology, polarity, motility, growth, and proliferation compared to those in their native 3D environments [51, 52].

2.5D culture systems serve as intermediates between 2D and fully embedded 3D cultures by introducing structural complexity through the inclusion of fibronectin, collagen IV, and poly-L-lysine while preserving free cell surfaces. Nanoscale engineering has demonstrated the influence of ECM topography on cellular behavior; for example, topographic features such as grooves, pits, and ridges influence cytoskeletal organization, focal adhesion dynamics, and mechanosensitive signaling. By modifying the tissue-level curvature while maintaining distinct apical and basal properties, 2.5D systems provide a physiologically realistic microenvironment that reflects ECM-mediated regulation more closely than conventional 2D cultures [53].

Fully 3D culture systems, particularly those utilizing OrmoComp®, PDMS, PEG, PAA hydrogels, and scaffold-based matrices, provide cellular microenvironments that better approximate in vivo conditions by increasing cell–matrix interactions and supporting tissue-like architecture. Matrix-hydrogel-based 3D models can provide an environment where cells interact dynamically with both the ECM and neighboring cells, as they resemble native tissue architecture. Studies have demonstrated that cells cultured in 3D environments exhibit physiological traits similar to those observed in vivo [54–56], including survival rates and responses to RT and chemotherapy [57]. Conventional approaches employ 2.5D and 3D models with ECM-coupled PAA gels, whose stiffness can be adjusted via photopolymerization or enzymatic degradation [58–61]. Additionally, hydrogel-based 3D culture systems are widely used in in vitro mechanobiology research because they allow cells to interact with their surroundings in a manner that resembles their native environment [51, 52, 62].

Recent advances have focused on generating 3D substrates with spatial gradients in stiffness or biochemical composition to better mimic the heterogeneity of living tissues. These gradients are typically established in gels derived from natural or synthetic materials, such as styrenated gelatin or collagen I, by controlling polymerization. Physical methods, including wedge-shaped gel [43] formation and component diffusion [63], have been explored; however, most modern approaches employ photoirradiation to modulate prepolymer cross-linking or induce controlled degradation of photosensitive polymers. Furthermore, 3D culture systems can be classified according to spatial scale, ranging from macroscopic constructs to micro- and nanoscale platforms such as organ-on-a-chip and microfluidic devices. These microengineered models enable fine control of mechanical and biochemical cues, although challenges persist in fabrication complexity, reproducibility, and the incorporation of physiologically relevant cell types [44, 64, 65].

### Microfluidic channel systems

Microfluidic channel systems enable cell culture under precisely controlled microenvironments, typically using channels 100–1000 µm wide to manipulate small liquid volumes within micrometer-scale networks. These systems provide high-throughput integrated platforms that operate at biologically relevant scales [44] and have been widely applied in tissue engineering, diagnostics, drug screening, cancer research, and stem cell biology. Their advantages arise from microscale physical phenomena, including high surface-area-to-volume ratios that enhance thermal uniformity and laminar flow that enables stable concentration gradients and fine control of soluble factors through diffusion-based mass transport [66, 67]. This controlled environment allows for the reproducible and quantitative analysis of cellular populations. Moreover, the design flexibility of microfluidic platforms permits the engineering of custom channel geometries and connectivity patterns that mimic in vivo architectures, supporting the development of microtissues and organ-on-a-chip models that replicate vascular [68], cardiac [69], and pulmonary structures [70]. These systems also provide precise control over cell arrangement, intercellular spacing, and cell–ECM interactions, thereby improving the physiological relevance of in vitro models.

PDMS is a commonly used material because of its ease of molding, biocompatibility, gas permeability for cell respiration, and optical transparency with low autofluorescence, making it suitable for live cell imaging [44]. Microfluidic devices have various structural complexities, including simple single-channel and single-layer configurations [71], multichannel arrangements within a single layer, often incorporating features such as gaps or posts for controlled substance exchange [72], and multichannel, multilayer architectures, where cells in one layer can interact with substances perfused through another [45].

Microfluidic systems allow small liquid volumes to be manipulated within micrometer-sized channels and offer high-throughput integrated environments operating at biologically relevant scales [44]. Their ability to provide environmental control and physiological mimicry has led to their use in tissue engineering, diagnostics, drug screening, fundamental cancer research, and stem cell studies.

### Advantages and limitations of 3D ECM environment

Cells within tissues interact dynamically with neighboring cells and the ECM, regulated by biochemical and mechanical factors. These interactions form a 3D communication network that supports tissue-specific architecture and homeostasis. Cellular processes such as proliferation, migration, and apoptosis are controlled by microenvironmental factors [73] (Table 1). Consequently, physiological cell–cell and cell–ECM interactions are more closely recreated by 3D cell culture systems than by 2D cultures. These aspects have made 3D cultures useful tools in fields including tumor biology, cell adhesion, migration studies, and epithelial morphogenesis [64].

Evidence suggests that 3D culture models can represent the native microenvironment of cells within tissues. Unlike 2D cultures, which impose constraints on cellular architecture and interactions, 3D systems closely reflect in vivo conditions by preserving spatial relationships and physiological responses. Cells cultured in 3D environments exhibit morphological and functional characteristics that differ from those of their 2D counterparts. The additional dimensionality of 3D cultures influences cell surface receptor organization, mechanical constraints, and intracellular signaling pathways, thereby modulating the gene expression and cellular behavior. These factors make 3D cultures more physiologically relevant, with observed cellular responses closer to those observed in vivo.

In recent decades, considerable effort has been directed toward optimizing and expanding the application of 3D culture systems. Although hydrogel-based matrices are commonly used for 3D cell cultures, they often lack the spatiotemporal complexity of native tissues. Since the 1990s, microfluidic systems have provided dynamic control over environmental factors, enabling the creation of reproducible 3D microenvironments that better resemble physiological conditions [2]. These models have been implemented in drug discovery, cancer research, stem cell studies, and the engineering of functional tissues for transplantation. By providing a more physiologically relevant in vitro environment, 3D culture systems enable the investigation of complex cellular responses in microenvironments that resemble in vivo conditions [64].

## Reciprocal influence of ECM stiffness and cancer treatment

Many solid tumors are characterized by a fibrotic, stiff ECM that promotes tumor progression and reduces therapeutic efficacy [2, 11]. Increased tumor stiffness has been identified as a biomarker of poor prognosis [74, 75] and increased invasiveness [76]. Research indicates that TME stiffness can compromise the effectiveness of chemotherapy [77, 78], immunotherapy [79], and RT [80, 81]. Therapeutic interventions can reciprocally modulate ECM mechanics; for example, chemotherapy may decompress tumors and soften the matrix, whereas RT frequently induces fibrotic stiffening. This bidirectional relationship has led to the investigation of strategies such as the use of stromal-modulating agents or the timing of therapies to exploit transient reductions in stiffness. The development of such approaches relies on quantitative tools to monitor the spatial and temporal variations in ECM mechanics. In this section, we review the influence of cancer treatments on TME stiffness and the reciprocal interactions between ECM mechanics and therapeutic responses.

### ECM stiffening impairs cancer treatment efficacy

Research on ECM stiffness has often focused on breast, liver, pancreas, and prostate cancers due to their distinct mechanical microenvironments and links to disease progression (Table 2). These cancers provide models for investigating the association between ECM biomechanical properties and clinical outcomes. Increased ECM stiffness in breast cancer promotes tumor invasion, metastasis, and drug resistance via mechanosensitive signaling pathways, such as the YAP and EGFR pathways [82, 83]. Similarly, increased stiffness in hepatocellular carcinoma is correlated with increased tumor proliferation, altered vascularity, chemoresistance, and worse prognosis [84, 85]. Pancreatic ductal adenocarcinoma (PDAC) is characterized by a dense, stiff, desmoplastic ECM that drives EMT and resistance to chemotherapy agents such as gemcitabine [86, 87]. Research on prostate cancer has indicated that ECM stiffness influences androgen receptor signaling, facilitates EMT, and is associated with disease progression and therapeutic resistance [88, 89]. In addition to these cancers, the role of ECM stiffness has been studied in colorectal, lung, and ovarian cancers.

**Table 2:** Summary of recent studies on the role of extracellular matrix (ECM) stiffness across various types of solid cancers

Cancer type	Key effects of ECM stiffness
Breast	Promotes tumor invasion, metastasis, and resistance via mechanosensitive pathways (YAP, EGFR) [82, 83]
Liver	Associated with tumor growth, altered blood vessel formation, and resistance to chemotherapy. Prognostic effect via ECM gene markers [84, 85]
Pancreas	Drives EMT and chemoresistance, and influences signaling pathways in a dense stroma [86, 87]
Prostate	Modulates androgen receptor signaling, promotes EMT, and correlates with tumor progression and advanced disease [88, 89]
Colorectal	Increases metastasis and tumor proliferation through EMT regulated by HSF4 signaling [90]
Lung	Stiffness regulates PD-L1 expression, which leads to evasion of the immune system and tumor growth [91]
Ovarian	Activates STAT3 mechanotransduction signaling, influencing tumor migration and adhesion molecules [92]

### Chemotherapy-induced modulation of ECM stiffness and reciprocal feedback mechanisms

Chemotherapy involves the use of cytotoxic drugs to kill rapidly dividing cells. Chemotherapeutic agents can induce cancer cell death by inhibiting mitosis, damaging DNA, and disrupting cellular metabolism [2]. Chemotherapy can shrink the tumor mass, but this cytoreduction can exert complex effects on the surrounding ECM. Tumor cell death due to chemotherapy can reduce mechanical stress and stiffness in the TME by decompressing the tissue. For example, platinum therapy in a pancreatic cancer model decreased tumor stiffness, and a favorable response to chemotherapy was associated with a softer ECM [11]. In triple-negative breast cancer, treatment with neoadjuvant chemotherapy reduced tumor stiffness as measured by MRE, and this reduction correlated with a better treatment response [93].

Conversely, chemotherapy can increase local ECM stiffness when the wound-healing response is prominent. In a mouse study, high-dose paclitaxel increased liver collagen V and realigned collagen I fibers, resulting in a pro-invasive ECM, as aligned, densely cross-linked collagen is stiffer than that in untreated controls [94]. A prospective breast cancer study applied shear-wave elastography (SWE) before and after neoadjuvant chemotherapy and analyzed collagen levels and architecture in resected specimens. Tumors with poor pathological responses retained high stiffness after treatment, and this residual elasticity correlated with the collagen fiber architecture, showing that chemotherapy-induced fibrosis rather than remaining tumor cells was responsible for the stiffer lesions [95]. These findings indicate the context-dependent and temporally dynamic nature of chemotherapy effects on the TME, which can either reduce or increase biomechanical barriers depending on treatment efficacy and timing.

Serial stiffness imaging has been investigated as a biomarker for chemotherapy-specific responses. In invasive breast cancer, SWE showed that a ≥35% decrease in the mean shear-wave velocity after two cycles of neoadjuvant chemotherapy predicted a complete pathological response with an AUC of 0.89, whereas tumors that remained stiff were typically nonresponders [96]. A subsequent multicenter study confirmed that an early (week 3) decrease in SWE-measured stiffness independently predicted chemotherapy response and overall survival [97]. In colorectal cancer liver metastases, a ≥13% reduction in SWE-measured stiffness at 4 weeks after systemic chemotherapy identified nonresponders with 97% sensitivity and was independently correlated with a longer progression-free survival [98]. These data suggest that monitoring stiffness during chemotherapy can identify sensitive tumors early, whereas rigid lesions may require alternative treatments or stroma-modulating agents.

The pretreatment stiffness of a tumor can predict its response to chemotherapy. Clinical studies of breast cancer using SWE have found that stiff tumors are correlated with chemoresistance, whereas more elastic tumors exhibit better responses [96, 99]. One explanation for this is that a dense, collagen-rich stroma reduces drug penetration and creates a tumor cell microenvironment. Consistent with this, in vitro experiments have shown that stiff matrices can protect cancer cells from chemo-induced apoptosis. Exposing MDA-MB-231 cells to doxorubicin on 10-, 38-, and 57-kPa substrates showed stiffness-dependent chemoresistance, with cell viability increasing with substrate elasticity [100]. In contrast, MCF-7 human breast cancer cells displayed the opposite trend: after exposure to identical doses of cisplatin or paclitaxel on matrices with stiffnesses of 5.3, 46.7, and 2710 kPa, their viability decreased as the stiffness increased, indicating greater drug resistance on softer substrates [101]. Similarly, the viability of SKOV-3 cells after exposure to 1 µM cisplatin was higher on a 0.5-kPa matrix than on a 25-kPa matrix [102]. Therefore, the relationship between matrix stiffness and chemosensitivity can be context-dependent; excessive stiffness hinders drug delivery, whereas very low stiffness can result in dormant cells evading therapy [11].

### Radiotherapy-induced modulation of ECM stiffness and reciprocal feedback mechanisms

RT uses ionizing radiation to kill cancer cells by damaging DNA [103]. The sensitivity of tumor cells to radiation is influenced by ECM stiffness via mechanotransduction pathways. A stiff ECM promotes the clustering of β1 integrins, activating FAK, integrin-linked kinase, and downstream PI3K/Akt and MAPK pathways [104, 105]. These signals increase cell survival and proliferation, counteracting radiation-induced apoptosis [106, 107]. Radiation can further increase integrin/FAK signaling, thereby strengthening the protective feedback mechanisms. Inhibiting β1 or αvβ3 integrins increases radiosensitivity [108–110], indicating the role of cell–ECM interactions in radiation responses. Hypoxia induced by a stiff ECM also mediates radioresistance. A dense, fibrotic ECM compresses the vasculature, causing chronic hypoxia, stabilizing HIF-1α, altering tumor metabolism and angiogenesis, and increasing cell quiescence [111–113]. Normalization of ECM stiffness can mitigate hypoxia-induced radioresistance by improving perfusion and oxygenation [114].

One approach involves targeting stromal components to increase the tumor sensitivity to RT. A stiff ECM and activated fibroblasts protect tumor cells from radiation. Consequently, drugs that inhibit CAFs or disrupt matrix cross-linking may increase the vulnerability of cancer cells to radiation. The death of tumor cells induced by RT can reduce tumor volume and edema, which might temporarily lower solid stresses, similar to the effect of cytotoxic agents. This could result in a tumor appearing softer upon palpation or imaging immediately after therapy [115–117]. However, ionizing radiation also induces a wound-healing response in normal and stromal tissues. Fibroblasts exposed to RT and RT-damaged cells receive signals, including TGF-β1, which induce them to differentiate into myofibroblasts, leading to increased deposition of collagen and ECM components [118]. TGF-β is a regulator of radiation-induced fibrosis, and its levels increase rapidly after RT, maintaining fibroblast activation and CTGF expression via a positive feedback loop [119, 120]. Consequently, RT can lead to tissue scarring months after exposure, causing the ECM to become denser and contract. Clinically, this results in organ fibrosis (e.g., pulmonary fibrosis after thoracic RT and liver cirrhosis after hepatic RT), which is dose-limited late toxicity [121].

Proton and carbon-ion therapies offer more targeted dose deposition, potentially sparing the normal tissue stroma surrounding tumors [122]. Preclinical studies have suggested that high-LET carbon ions may induce less TGF-β release and fibroblast activation than X-rays [123, 124]. There is no clinical evidence that carbon-ion RT causes more fibrosis than photon-based RT at equivalent doses [125]. Moreover, carbon ions are less oxygen-dependent (lower OER), allowing them to maintain their effectiveness in hypoxic, stiff tumors that are more resistant to X-rays. These findings suggest that particle therapy may partially overcome the stiffness-related radioresistance. However, within the high-dose Bragg peak region [126], particles damage stromal cells and can trigger fibrosis if normal tissues are present in the irradiated field. The net effect on ECM remodeling depends on treatment planning, specifically the volume of normal stroma irradiated, and the biological responses. One study observed that carbon-ion RT did not increase TGF-β1 and interleukin (IL)-10 (immunosuppressive and profibrotic cytokines) as much as X-rays did [124, 127], potentially leading to a less-immunosuppressive TME after treatment. Overall, particle therapy may reduce collateral fibrotic damage. However, supplementary strategies are required to address ECM stiffness in tumors with fibrosis.

In summary, ECM stiffness modulates radiosensitivity via integrin/FAK signaling, mechanotransduction pathways, DNA repair efficacy, and hypoxia generation. While increased ECM stiffness can reduce the efficacy of RT against tumor cells, RT itself also affects the normal tissues surrounding the irradiated field.

### Immunotherapy–ECM interactions: variability in stiffness modulation and reciprocal effects

Immunotherapy does not directly target stromal cells or the ECM; its effects on ECM stiffness are mostly indirect consequences of immune activity [11]. ECM stiffness influences the immune response more than the immune response influences the ECM stiffness. A stiff, desmoplastic tumor can act as both a physical and biochemical barrier [114]. Dense collagen bundles increase interstitial pressure and impede T-cell movement through tissue [128–130]. Additionally, a rigid ECM may promote immune evasion mechanisms; studies have shown that increased matrix stiffness upregulates checkpoint proteins such as PD-L1 in cancer cells. For example, lung cancer cells cultured on stiffer substrates express higher levels of PD-L1, which inhibits CD8^+^ T cells and reduces immune activity [91, 131]. High collagen density in tumors is associated with resistance to PD-1/PD-L1 blockade [132, 133]. These factors result in the direction of causality typically being from ECM stiffness to immunotherapy effectiveness rather than the reverse.

When immunotherapy leads to tumor eradication, it may also lead to ECM remodeling. Infiltrating cytotoxic T cells and M1-polarised macrophages secrete MMPs and other proteases that degrade ECM components, potentially softening the tumor stroma [10]. Inflammatory macrophages (with M1 polarization) create an initial proteolytic milieu via MMP-9 and MMP-12 that softens dense collagen and elastin networks [134, 135]. Incoming cytotoxic T cells then add granzyme B, which further degrades specialized ECM components such as basement membranes and fibrillin microfibrils [136, 137]. These combined actions accelerate matrix turnover, facilitating immune cell infiltration but also risking collateral tissue damage, a factor in chronic inflammation, tumor progression, aneurysm formation, and nonhealing wounds [138]. In melanoma, clinical responses to immunotherapy are linked to matrix degradation in areas that promote T-cell migration [139, 140]. Alternatively, if the immune response results in a chronic inflammatory but noncytolytic state, the ECM can become stiffer. M2-polarised macrophages and certain T-cell subsets release TGF-β and IL-13 [141], which stimulate fibroblasts to deposit collagen, leading to fibrosis. This phenomenon is observed in tumors where immune cells surround the periphery but cannot penetrate the center; the peritumoral stroma becomes a fibrotic “immune exclusion” zone [142–144].

### Mechanobiology-driven therapeutic strategies: matrix modulation across cancer treatment modalities

Designing therapeutic strategies based on tumor mechanobiology requires the integration of both the treatment modality and its underlying biomechanical mechanisms, particularly how each intervention modulates tumor stiffness, ECM architecture, and biophysical signaling. In this section, we first outline the shared core ECM-associated therapeutic targets of chemotherapy, RT, and immunotherapy. We then discuss therapy-oriented, modality-specific extracellular matrix remodeling strategies, highlighting how matrix modulation can enhance therapeutic efficacy and overcome resistance in each treatment context.

#### Targeting shared core mechanosignaling pathways

*Targeting Stromal Reprogramming *via* TGF-β Inhibition:* TGF-β, a key cytokine linking ECM stiffness to immune exclusion, activates fibroblasts to deposit collagen while suppressing T-cell activity [133, 145]. In PDAC, losartan-mediated inhibition of TGF-β signaling in CAFs has been translated into clinical evaluation (phase II trial, NCT01821729) [146], underscoring the therapeutic potential of targeting stiffness-associated stromal pathways. Combining anti-TGF-β agents with checkpoint blockade alleviates stiffness-induced immune evasion and enhances antitumor immunity. The bifunctional antibody M7824 (bintrafusp alfa), which simultaneously blocks PD-L1 and TGF-β, suppresses SMAD2/3 signaling in CAFs, reduces collagen deposition, and increases intratumoral CD8^+^ T-cell infiltration, achieving superior tumor control compared with PD-L1 monotherapy [147].

*Targeting Mechanotransduction *via* FAK/Integrin Inhibition:* Inhibiting FAK or its upstream integrins disrupts the mechanotransduction pathways through which ECM rigidity propagates protumorigenic signaling to cancer and stromal cells [9]. In desmoplastic PDAC, FAK activation in CAFs induces CXCL12, CCL5, and PD-L2 expression, driving myeloid recruitment and T-cell exclusion [148]. FAK blockade remodels the collagen-rich stroma into an immune-permissive microenvironment, enhancing anti-PD-1 responsiveness [149]. Clinically, defactinib combined with pembrolizumab achieved stromal p-FAK suppression, reduced CAF signatures, and produced early objective responses in refractory PDAC [150]. Similarly, FAK inhibition in PDAC models counteracts fibroblast-mediated radioresistance and improves tumor cell death when combined with RT [151, 152].

#### Therapy-oriented ECM remodeling strategies

**Fig. 2:**
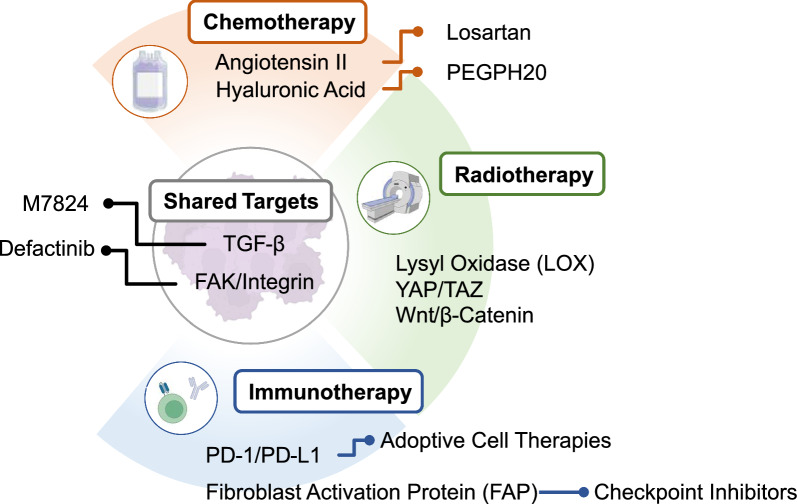
Mechanobiology-driven therapeutic strategies across cancer treatment modalities converge on shared core signaling pathways that regulate ECM organization and tumor biomechanics. Central among these are TGF-β signaling and FAK/integrin-mediated mechanotransduction, which collectively orchestrate ECM remodeling, tumor stiffness, and stromal–immune interactions. In parallel, chemotherapy, radiotherapy, and immunotherapy act through distinct upstream mechanisms and engage therapy-specific ECM remodeling processes, as detailed in this article. Together, these shared and modality-specific mechanisms shape the tumor microenvironment, influencing drug delivery, immune cell infiltration, and overall treatment response

Therapy-oriented ECM remodeling strategies target the physical and biochemical properties of the TME to improve the efficacy of standard cancer treatments by addressing both modality-specific mechanisms and shared mechanotransduction pathways (Fig. 2).

*Chemotherapy:* Research has explored softening the tumor matrix to enhance drug penetration using stromal modulators such as the angiotensin II receptor blocker losartan [153, 154]. Another agent, PEGPH20 (pegylated hyaluronidase), targets hyaluronic acid in the stroma, reducing interstitial fluid pressure and stiffness [155]. In PDAC models, losartan pretreatment lowers solid stress and vascular pressure, thereby improving chemotherapy delivery and efficacy [154]. A phase II trial combining neoadjuvant FOLFIRINOX with losartan in locally advanced PDAC is currently underway [146].

*RT:* Inhibiting LOX during RT may prevent matrix stiffening and reduce metastasis [156]. Although not yet widely tested clinically, preclinical studies suggest that LOX inhibition preserves tumor elasticity and enhances RT efficacy [91, 157, 158]. The combination of pentoxifylline, a hemorheologic agent that enhances blood flow, with vitamin E has been shown to mitigate radiation-induced fibrosis, particularly in the breast and head-and-neck regions, by softening fibrotic tissue [159–161]. Other mechanosensitive pathways, such as YAP/TAZ and Wnt/β-catenin, are activated by a stiff ECM and contribute to radioresistance through survival and DNA repair genes [162–165]. Conversely, softer matrices delay double-strand break repair and enhance radiosensitivity. Notably, FAK inhibition in head and neck cancer cells suppresses both homologous recombination and nonhomologous end-joining repair [59, 157], thereby amplifying RT-induced DNA damage [166].

*Immunotherapy:* Enzyme-armed immune cells are engineered to overcome ECM-associated barriers. For example, CAR T-cells have been modified to secrete heparanase, which degrades matrix proteoglycans and enhances T-cell infiltration into dense tumors [167]. Other strategies include targeting the fibroblast activation protein on CAFs to remodel the stroma [168–170], or engineering CAR T-cells to locally release collagenase or proinflammatory cytokines such as IL-12 [171], thereby improving immune access within dense tumor matrices. These approaches, primarily evaluated in preclinical models, aim to enhance the immune cell function by mitigating ECM-mediated resistance.

Taken together, chemotherapy, RT, and immunotherapy interact reciprocally with tumor mechanobiology, but through different mechanisms [172–174]. Chemotherapy and RT can both transiently reduce tumor stiffness through cytoreduction and tissue decompression, while also promoting secondary matrix stiffening when treatment-associated injury activates wound healing and fibrotic stromal responses [172, 175]. This fibrotic remodeling is particularly well established after RT, in which TGF-β-driven fibroblast activation contributes to delayed tissue scarring and long-term ECM stiffening [121]. In contrast, immunotherapy is more strongly influenced by pre-existing matrix stiffness, which shapes immune cell infiltration and therapeutic efficacy [128, 172, 173, 176]. Effective immune-mediated tumor clearance may promote ECM degradation through protease-associated remodeling, whereas ineffective or chronic inflammatory responses may enhance fibrosis and immune exclusion [174, 177, 178]. Thus, all three modalities share a dynamic interplay with ECM remodeling but differ in the directionality and mechanisms of their mechanobiological effects [172].

### Therapeutic trade-offs and context-dependent risks of matrix-targeting therapies

Matrix-targeting therapies can enhance drug delivery, normalize tumor stroma, and improve treatment sensitivity by reducing ECM-mediated physical and signaling barriers. However, due to the context-dependent roles of ECM components and therapy-induced remodeling, these interventions may also promote invasion, resistance, and immune escape. Therefore, precise targeting, biomarker-guided selection, and rational combinatorial strategies are required.

*Adverse paradoxical effects*: Despite the benefits of matrix-targeting therapies, ECM perturbation may generate pro-tumor effects due to its dual structural and signaling roles. Matrix degradation or remodeling can remove physical constraints and expose pro-migratory cues, thereby enhancing invasion and metastasis in certain contexts [172]. Importantly, chemotherapy-induced ECM remodeling may result in opposite biomechanical outcomes. While reduced tumor cellularity and interstitial pressure can transiently soften the matrix and improve drug penetration, therapy-induced tissue injury may also activate compensatory wound-healing responses involving myofibroblast activation, TGF-β signaling, and collagen crosslinking, ultimately promoting fibrosis and recurrent tissue stiffening following RT or high-dose chemotherapy [121]. Broad-spectrum MMP inhibition has also failed clinically, partly due to functional heterogeneity and antitumor roles of specific MMPs [177]. Moreover, conventional therapies remodel flexible ECM environments that may induce reversible tumor cell quiescence, promoting resistance to cytotoxic therapies and later metastatic relapse [172]. ECM composition and stiffness also regulate immune infiltration; thus, ECM alteration may create immune-suppressive microenvironments or reduce immune access [172]. Enzymatic degradation strategies may introduce trade-offs by interfering with other modalities, highlighting the need for controlled remodeling [179].

*Challenges in clinical translation*: Clinical translation of matrix-targeting therapies remains limited by tumor heterogeneity and ECM complexity. Broad-spectrum MMP inhibitors have largely failed due to off-target effects [177], while integrin- and ECM sensor-targeted therapies show limited efficacy because of redundancy in mechanotransduction signaling [173]. Although hyaluronidase-based systems, nanocarriers, CAF-targeting approaches, and combinatorial nanoplatforms have demonstrated strong preclinical antitumor activity [173, 179], robust clinical validation remains lacking. Furthermore, while anti-angiogenic strategies aimed at vascular normalization may improve drug delivery, their ECM-related clinical benefits remain unclear.

## Principles of elasticity measurements

Quantitative assessments of tissue stiffness are fundamental for understanding the biomechanical influences of the ECM and for developing therapeutic strategies. The quantification of stiffness relies on the measurement of elastic moduli, which describe how a material deforms in response to applied forces. These moduli were measured and interpreted using physical models that depend on the material’s geometry and the nature of wave propagation, primarily distinguishing between bulk-wave and guided-wave models (Table 3).

**Table 3:** Comparison of bulk waves and guided waves in elasticity measurements

	Bulk waves	Guided waves
Propagation medium	Unbounded (effectively infinite)	Bounded (dimensions comparable to or smaller than the mechanical wavelength)
Common wave types	Compressional waves, shear waves	Surface waves (e.g., Rayleigh waves), Lamb waves
Dispersion	Low	High
Wave velocity	Dependent on material properties (e.g., moduli, density)	Dependent on frequency, material properties, and geometry (e.g., thickness, boundary conditions)

### Tissue stiffness and bulk wave elastography

In standard elasticity imaging, large organs are treated as effectively unbounded media, allowing mechanical waves to propagate as bulk waves. When force is applied to a tissue, its mechanical response is governed by its resistance to different types of deformations. Two primary physical properties are considered for biological soft tissues:

*Bulk Modulus (*$K$*)*: The resistance to a change in volume under pressure. Because soft tissues are primarily composed of water, they are nearly incompressible. Consequently, the bulk modulus remains relatively uniform across almost all soft tissues.

*Shear Modulus* ($G$): The resistance to a change in shape without a change in volume. Soft and flexible tissues easily undergo shear deformation.

*Young’s Modulus* ($E$): The resistance to linear tension or compression along a single axis. This is the most frequently reported metric in mechanobiology when discussing the stiffness of cell culture substrates or ECM in kilopascals.

From a biological and oncological perspective, $G$ is highly relevant. While $K$ changes very little between healthy and diseased states, the shear modulus can span several orders of magnitude depending on the tissue type, ECM remodeling, and pathology. For example, the dense, cross-linked ECM of a solid tumor exhibits a significantly higher shear modulus than the surrounding healthy tissue [180].

Because of this dramatic contrast, wave-based imaging techniques in soft tissues primarily utilize shear waves. By observing the speed at which shear waves travel through a tissue, researchers can directly estimate its overall stiffness, where mechanical waves travel faster through stiffer materials. By mapping these wave speeds, clinicians can generate a spatial map of tissue stiffness, providing a robust physical biomarker for identifying tumors, fibrosis, and abnormal ECM dynamics.

### Guided waves in bounded media

The assumptions of bulk-wave elastography work well for large organs like the liver and breast. However, when the dimensions of a tissue are thin or layered and comparable to the wavelength of the mechanical wave, these assumptions fail. This geometric confinement is highly relevant for structures such as the cornea, thin blood vessel walls, skin layers, and small biological samples housed within microfluidic channels used in in vitro cancer research [181–183].

In these bounded structures, mechanical waves bounce off the tissue borders and propagate as guided waves. Unlike bulk waves, which travel at a constant speed regardless of frequency, guided waves are dispersive. This implies that the speed of the wave changes depending on its frequency.

Because of this dispersion, tissue stiffness cannot be calculated from a single simple wave speed measurement. Instead, researchers must measure how wave speeds change across a wide range of frequencies to create a dispersion curve. By matching this measured curve to theoretical physical models, such as Lamb wave models [184], which describe how waves behave in thin, plate-like materials, researchers can accurately extract the underlying stiffness of tissue. While more complex to analyze, recognizing and measuring guided waves is essential for accurately evaluating the mechanical properties of thin anatomical structures and microscopic oncology models, where boundaries dictate physical behavior [182, 185–188].

## Elasticity measurement techniques

Various techniques are available for measuring the mechanical properties that govern tissue stiffness. These methods can be broadly categorized based on their primary physical principles, such as those relying on shear-wave propagation versus other mechanical or optical interactions. Each method has specific characteristics regarding spatial resolution, imaging depth, assessed modulus, and limitations (Table 4).

**Table 4:** Summary of techniques for measuring tissue mechanical properties

Elasticity imaging methodology	Principle	Spatial resolution	Temporal resolution	Exogenous agent	Handle optical turbidity*	Measuring time	Elasticity measurement	Internal heterogeneity assessment	Best use case
MRE [189, 190]	Mechanical wave generation & MRI tracking	mm	Seconds to minutes	Yes	Yes	Minutes	$G$	No	In vivo whole-organ stiffness monitoring and deep-tissue tumor diagnosis
µMRE [189, 191]	MRE using higher-frequency waves	µm	Minutes	Yes	Yes	Minutes	$G$	Yes	Long-term nondestructive monitoring of 3D cultures and tissue constructs
OCE [192–194]	Displacement tracking during applied stresses	µm	Seconds	No	No	~10 s	$G$	Yes	High-resolution mapping of near-surface layered tissues and tumor margins
Optical SWEI [195, 196]	Shear-wave generation using pulsed laser irradiation	µm	Seconds	No	No	Seconds	$G$	Yes	Ultrafast internal mapping of large single cells (e.g., oocytes) and embryos
LSCI-SW [197]	Laser-speckle-contrast SWEI	Sub-mm	Seconds	No	No	Minutes	$G$	Yes	Long-term spatiotemporal monitoring of 3D TME
US SWEI [198]	SWEI with an array US transducer	mm	Seconds	Yes	Yes	Seconds to minutes	$G$	Yes	Real-time clinical focal lesion characterization and biopsy guidance
Single-element US SWEI [199]	SWEI with a single US transducer	–	Seconds	Yes	Yes	~10 s	$G$	No	Longitudinal monitoring of bulk stiffness in 3D cultures
AFM [200]	Force-produced microscale or nanoscale indentations using cantilevered tips	µm	Hours	No	Yes	Hours	$E$	No	High-resolution single-cell mechanobiology and surface mechanics of ECM
Shear rheology [201]	Strain responses to applied torques	–	–	No	Yes	Seconds	$G$	No	Bulk mechanical characterization and tuning of macroscopic biomaterials
Particle-tracking microrheology [202, 203]	Microscopic motions of fluorescent particles	Sub-µm	Seconds	Yes	No	10–20 s	$G$	No	High-speed mapping of internal subcellular mechanics and dynamic cell responses
Brillouin microscopy [204]	Brillouin frequency-shift tracking	Sub-µm	Seconds	No	No	Hours	$K$	Yes	High-resolution 3D mapping of tumor spheroids and noncontact single-cell analysis

*“No” indicates a restricted imaging depth

*MRE* magnetic resonance elastography, *MRI* magnetic resonance imaging, *OCE* optical coherence elastography, *SWEI* shear-wave elasticity imaging, *US* ultrasound, *AFM* atomic force microscopy

### Shear-wave-based approaches

Techniques based on shear waves rely on generating and detecting shear waves within a material, with the shear-wave speed (SWS) being directly related to $G$.

**Fig. 3:**
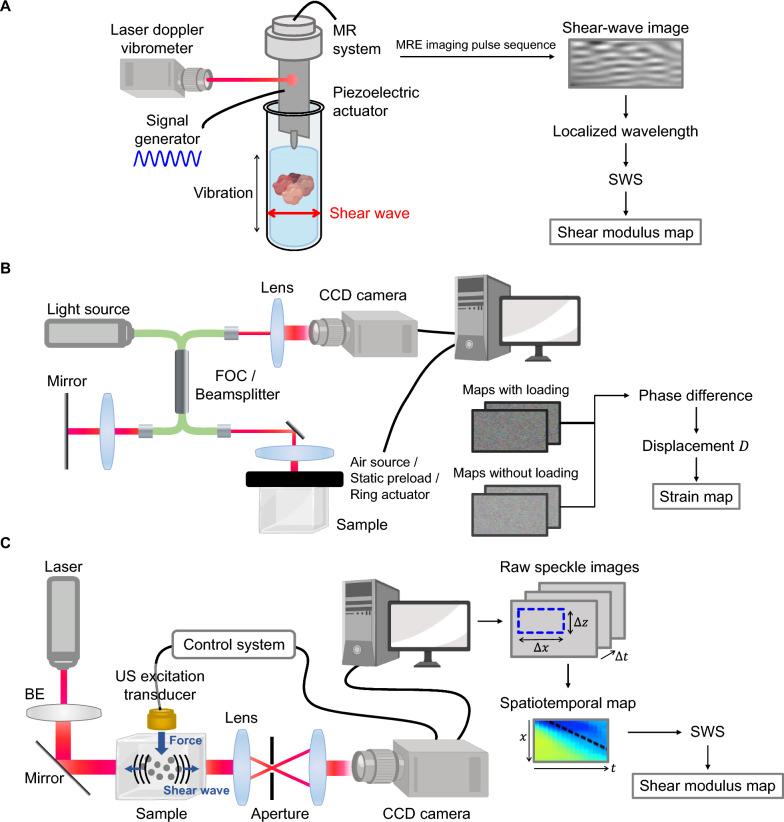
Schematics of selected elasticity measurement techniques. **A** Microscopic magnetic resonance elastography. **B** Optical coherence elastography. **C** Laser-speckle-contrast shear-wave imaging. *FOC* fiber optic coupler, *BE* beam expander, *US* ultrasound, *ROI* region of interest, *SWS* shear-wave speed

*MRE*: Noninvasively quantification of $G$. MRE uses external actuators to generate low-frequency shear waves in the tissue, whereas phase-contrast MRI sequences are used to simultaneously map the resulting microscopic tissue displacements [205–207]. These displacement maps can be analyzed to determine the wavelength of the shear waves and SWS, from which $G$ is calculated. MRE was initially developed for clinical whole-organ diagnostics, such as identifying liver or mapping deep brain tissue [205]. Subsequent modifications have led to microscopic MRE (µMRE) to achieve submillimeter resolution, which is ideal for the long-term, nondestructive monitoring of 3D tissue-engineered constructs [190, 191, 208] (Fig. 3A). However, inducing the required high-frequency mechanical vibrations in very small (subcentimeter scale) or stiff biomaterials remains a technical challenge for µMRE [189].

*Optical coherence elastography (OCE)*: A high-resolution technique for mapping tissue mechanics based on optical coherence tomography (OCT), which produces microstructural images from the analysis of backscattered light [209]. OCE operates on two primary principles: (1) quasistatic strain imaging and (2) dynamic wave propagation. Strain-based OCE involves applying slow quasistatic compression to the tissue, with OCT used to track the subsurface speckle patterns arising from the interference of light scattered by the tissue microstructure. Displacements were quantified from the phase differences, and the resulting strains were calculated from these displacement fields. The relationship between the strain and applied stress corresponds to $E$ [210, 211] (Fig. 3B). In contrast, wave-based OCE applies a transient mechanical force (often from an air puffer or small mechanical actuator) to generate propagating shear or guided waves [186, 212], with OCT used to track the spatiotemporal characteristics of wave propagation. These data were used to calculate the wave speeds and produce maps of $G$.

OCE enables 3D imaging of the elastic properties of a sample and can be used to evaluate near-surface layered tissues in vivo, assess microscopic tumor margins, and monitor the skin [186, 213]. OCE is frequently used in ophthalmology to measure corneal mechanical properties due to its high resolution and sufficient penetration depth in transparent ocular tissues. In this application, the thin layers of the cornea result in mechanical waves propagating as guided waves. These waves are dispersive, meaning that their phase velocity changes with frequency. Therefore, to determine the corneal elasticity, the experimentally measured phase velocity dispersion data needs to be fitted to theoretical Lamb wave models. One approach is to use a model that assumes the cornea to be a transversely isotropic material, in which its mechanical properties vary along its surface versus through its thickness. This fitting process allows multiple elastic constants to be estimated, providing a more comprehensive mechanical characterization than a single-modulus value [185, 212, 214].

*Optical shear-wave elasticity imaging (SWEI)*: Uses a focused laser pulse to rapidly heat a small volume within a sample to induce localized thermal expansion. This expansion acts as a mechanical impulse that generates shear waves. A separate optical system, often involving a high-speed camera or an interferometer, tracks the propagation of these shear waves. Measuring the travel time over a known distance allows the SWS to be determined, from which $G$ is calculated. Because it offers ultrafast internal mechanical mapping, optical SWEI has been utilized to investigate the highly dynamic cytoskeletal events of large single cells, such as oocytes and early embryos in vitro. Volumetric visualization has also been achieved using OCT C-scans for 3D optical SWEI [196, 215]. However, like many optical methods, light scattering usually restricts the penetration depth to around 200 µm [216]. Laser-induced thermal damage to collagen-based materials has also been reported [192].

*Laser-speckle-contrast shear-wave imaging (LSCI-SW)*: LSCI-SW is highly effective for label-free, long-term spatiotemporal monitoring of 3D tumor microenvironments and biomaterials [197]. This hybrid technique utilizes a US transducer to generate shear waves, whereas coherent laser illumination and speckle contrast analysis are used to track wave propagation (Fig. 3C). The propagation of the shear wave causes microscopic tissue motion that modulates the speckle pattern. Tracking the propagation of these motion-induced speckle changes allows the SWS to be mapped and hence $G$ calculated.

LSCI-SW has been useful for correlating the matrix fiber density with stiffness variations associated with cell proliferation over time in 3D cultures at submillimeter resolution [197, 216, 217]. The incorporation of tomographic reconstructions enables 4D (3D plus time) visualization of shear-wave wavefronts, which can be used for multidirectional stiffness measurements in both homogeneous and heterogeneous 3D culture substrates during ECM remodeling [218]. Despite its utility, LSCI-SW requires optical transparency, which restricts its application in optically dense tissues due to light scattering. Additionally, these analyses can be complicated by a decrease in speckle contrast when positive and negative axial movements superimpose during shear-wave propagation, making viscosity measurements challenging [216].

**Fig. 4:**
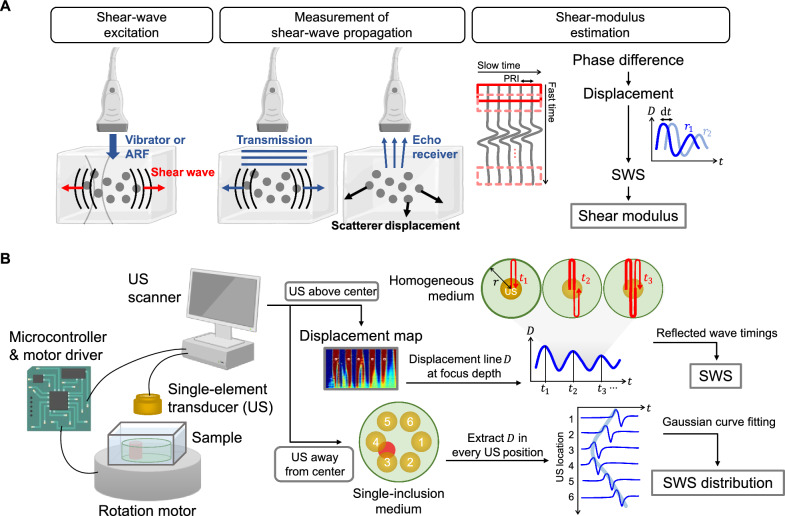
Schematics of US-based elasticity imaging systems. **A** Ultrasound shear-wave elasticity imaging. **B** Reflected shear-wave computed tomography. *ARF* acoustic radiation force, *PRI* pulse repetition interval, *SWS* shear-wave speed

*US SWEI*: A common technique for assessing elasticity. This noninvasive method can be used to quantify aspects of tissue stiffness and is mainly used to determine $G$. Shear waves are typically generated within the tissue by applying a short, intense, focused US pulse known as an acoustic radiation force or by using an external vibrator. This pulse created a localized transient tissue displacement, after which the transducer rapidly transmitted unfocused or plane wave pulses at very high frame rates. Raw radio frequency (RF) signals reflected from the tissue were captured. Comparing the phases of consecutive RF signals allows the microscopic tissue displacements induced by the propagating shear wave to be estimated. The speed of the shear wave travelling perpendicular to the initial displacement is then tracked [194, 198, 219–222] (Fig. 4A), from which $G$ can be calculated.

US SWEI is extensively used clinically for focal lesion characterization, targeted biopsy guidance, and noninvasive staging of diffuse fibrotic diseases [223–225]. However, standard clinical US SWEI typically operates at 2–10 MHz to optimize centimeter-scale penetration and therefore lacks the spatial resolution to study 3D cell cultures with millimeter-scale dimensions [222, 226, 227]. While techniques exist to measure anisotropy using rotating transducers [228] or achieve 3D visualization via mechanical or electrical scanning [229–231], they are often constrained by physical limitations associated with lower frequencies. Furthermore, conventional US SWEI often acquires data in the B-scan plane (perpendicular to the transducer face), whereas optical methods frequently operate in the C-scan plane (parallel to the transducer/objective face), which is better suited for layered 3D cultures [222].

One approach for adapting US SWEI to smaller scales uses a single-element transducer to generate and detect shear waves. This method is suited for the streamlined longitudinal monitoring of bulk stiffness in optically opaque 3D cell cultures and cell-induced matrix remodeling [199]. While useful for homogeneous materials, it is less suited for inhomogeneous samples because it only measures the average SWS. The findings of that study suggested that smaller transducers operating at higher frequencies could further reduce the sample size to improve agent diffusion in 3D cell cultures. US shear-wave computed tomography (SWCT) was developed to address inhomogeneities and provide 3D stiffness mapping. SWCT can reconstruct high-resolution 3D maps of stiffness, including C-scan SWS maps (related to $G$) that are suitable for characterizing complex and inhomogeneous samples [232]. The first SWCT systems used dual high-frequency transducers, whereas more recent SWCT approaches reconstruct inhomogeneous stiffness distributions in millimeter-scale 3D cell cultures using rotational scanning with a single-element transducer [233] (Fig. 4B).

A critical consideration when performing small-scale measurements is the possibility of sample boundaries influencing wave propagation, causing the measured SWS to deviate from the true bulk SWS. To validate the results, the SWS measurements made using these US methods were compared with those obtained using shear rheology, a standard technique for measuring bulk mechanical properties [226]. This cross-validation confirmed that the measured SWS reflects the $G$ of the material.

Despite these developments in sensitivity and resolution for small-scale systems, challenges remain in translating these methods into real-world applications. 3D cell cultures contain diverse structures and multiple cell types, and the associated complexity and biological variability can complicate the propagation and interpretation of shear waves, necessitating further optimization of these imaging techniques.

### Non-shear-wave-based approaches

Non-shear-wave-based techniques measure mechanical properties using methods that do not directly involve measuring the SWS, such as direct force application, analysis of intrinsic material responses to light, or tracking deformations under stress.

**Fig. 5:**
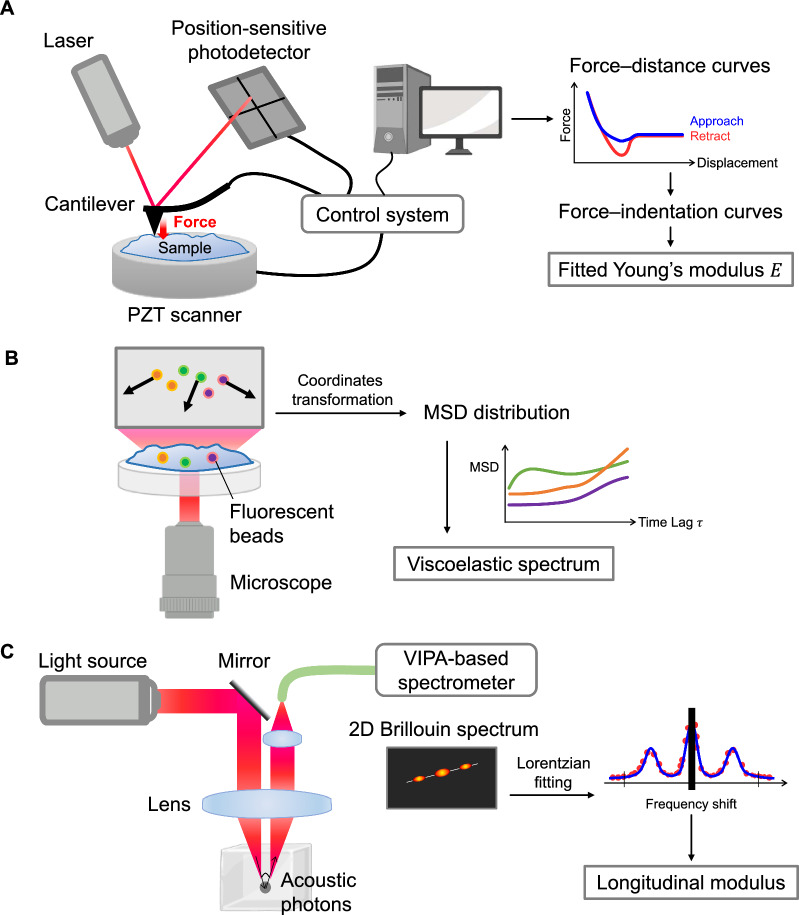
Schematics of non-shear-wave-based elasticity measurement systems. **A** Atomic force microscopy. **B** Particle-tracking microrheology. **C** Brillouin microscopy. *PZT* piezoelectric transducer, *MSD* mean squared displacement, *VIPA* virtually imaged phased array

*AFM*: Involves direct mechanical interactions with a sample, where a tiny cantilever with a sharp tip is used to create a microscale or nanoscale indentation on the sample surface. The relationship between applied force and the resulting indentation depth yields the local $E$ [200, 234] (Fig. 5A). AFM maps viscoelastic properties with microscale spatial precision, providing high-resolution single-cell mechanobiology and probing the localized surface mechanics of the ECM [235–237]. However, AFM mainly measures surface properties and hence might not represent bulk material properties or reveal internal differences within anisotropic samples [197, 238]. The measurement process can be slow, making it less suitable for imaging live, dynamically changing cells. Additionally, the localized nature of the indentation may not reveal the overall behavior of the material and can damage biological samples [200, 238].

*Shear rheology*: Applies periodic torsional forces to characterize shear viscoelasticity at different strain rates, providing macroscopic bulk mechanical characterization. It is the standard technique for designing and tuning biomimetic 3D tissue scaffolds [201, 239, 240]. While providing information about overall material behavior, the sample must be confined between plates or geometries. This confinement restricts the investigation of dynamic changes, such as during media exchange or long-term cell activity. This limitation is particularly relevant for the investigation of cell-driven ECM remodeling.

*Particle-tracking microrheology*: Estimates local viscoelasticity by analyzing the movement of microscale fluorescent particles (Fig. 5B), making it ideal for high-speed, nondestructive mapping of internal subcellular mechanics and dynamic cytoskeletal responses. In passive microrheology, the inherent thermal (Brownian) motion of particles is tracked using optical microscopy. The displacements of these particles are related to the local $G$ [202, 203]. Active microrheology involves applying external forces to particles, and their induced displacement is measured to infer the mechanical properties of the material [241, 242]. However, both methods require an unobstructed optical path to allow particle tracking, which restricts the imaging depth and size or thickness of the 3D cultures that can be analyzed [197].

A common problem for invasive methods such as AFM and rheology is that applied forces can disturb or damage soft 3D culture samples. This can complicate long-term studies and the interpretation of results.

*Brillouin microscopy*: Optically measures stiffness by analyzing frequency shifts in scattered laser light. This frequency shift (the Brillouin shift) reflects intrinsic thermal acoustic waves within a material. By measuring this shift using a high-resolution spectrometer, the longitudinal modulus (which is related to $K$) can be determined without applying external forces [204, 243] (Fig. 5C). Because it provides micrometer resolution in 3D, Brillouin microscopy serves as a tool for the noncontact mapping of intact tumor spheroids and in situ single-cell analysis. However, its penetration depth is restricted to a few hundred micrometers in scattering tissues due to light attenuation [244].

## Future directions and conclusion

Future therapeutic strategies should emphasize precision and combination approaches. Matrisome-guided targeting of specific ECM components may improve patient stratification and reduce off-target effects [173]. Key mediators include integrin/ILK signaling, autophagy, stress MAPKs, and CDK inhibitors. Targeting mechanotransduction pathways, including integrins and Piezo1/TRPV4 signaling, may provide an alternative to direct ECM depletion [25]. Combining ECM-targeting therapies with immunotherapy, chemotherapy, or vascular normalization may enhance efficacy while limiting pro-invasive remodeling [172]. In addition, tumor-targeted or activatable delivery systems may spatially restrict ECM remodeling and reduce systemic toxicity [179, 245, 246].

Developments in elasticity imaging are focused on enabling noninvasive 3D measurements of gel mechanics at the millimeter scale required for in vitro models. Optical techniques offer high spatial resolution, but their shallow penetration depth is a primary limitation. Hybrid approaches such as LSCI-SW address this by using a US transducer to generate deeper waves while retaining high-resolution optical tracking. Concurrently, US-based methods have been miniaturized for analogous applications, with high-frequency, single-element US SWEI and SWCT enabling the characterization of heterogeneous structures and the monitoring of temporal stiffness dynamics.

Measuring elasticity within microfluidic channels requires further miniaturization of transducers, drawing on technologies initially developed for medical applications such as intravascular US (IVUS) [229, 230, 247]. A challenge remains in the trade-off between spatial resolution and penetration depth. High-frequency (>30 MHz) transducers provide short wavelengths for high resolution but experience higher attenuation that reduces imaging depth. Conversely, lower frequencies (<10 MHz) penetrate deeper but lack the required resolution to visualize fine structures within microfluidic devices. Strategies adapted from IVUS, such as compact dual-element transducers with separate excitation and detection elements [231], have been used to estimate the stiffness of small structures such as ex vivo atherosclerotic arteries [231] and corneas [248]. In a typical setup, a low-frequency transducer induces shear waves, while a cofocused high-frequency transducer detects the resulting tissue displacement at an improved resolution. The SWS can then be estimated by relating the propagation distance to the measured time-to-peak displacement. However, uncertainty in the generation time of the shear waves can introduce inaccuracies in SWS calculations. Trielement transducer configurations have been proposed, where two receiving elements detect wave propagation over a known distance. This time-of-flight approach represents a potential pathway for developing high-resolution US elastography for evaluating biomechanical properties of microfluidic systems.

Current imaging methods provide quantitative data on matrix elasticity dynamics, but they still struggle to fully reconstruct elasticity maps across complex 3D environments mimicking the in vivo TME. ML techniques have been used to reconstruct inhomogeneous elasticity distributions within centimeter-scale 3D models. Deep convolutional neural network architectures have improved elasticity maps and the resolution of reconstructions from shear-wave propagation data, enabling the determination of shear modulus distributions and mechanical anisotropies in centimeter-scale biomaterials [249–251]. In US computed tomography, ML has successfully reduced long computational times and improved the accuracy of acoustic property mapping [252, 253]. Future studies should focus on validating and optimizing these ML approaches for complex biological structures to enable their application in tissue engineering and mechanobiology.

A key application of these advanced imaging techniques is understanding the role of ECM in therapeutic resistance. ECM stiffness can obstruct drug penetration, reduce the effectiveness of RT, and restrict immune cell infiltration. Current research integrates chemotherapy, RT, and immunotherapy to overcome these barriers. Concurrent chemoradiotherapy (CCRT) is a front-line treatment for several solid tumors and has shown survival advantages over sequential regimens [254–256]. CCRT modulates the TME by regulating ECM stiffness, contributing to improved clinical outcomes. For example, cytotoxic doublets can mitigate CAF activity [257], and radiation induces matrix-degrading proteases that lower collagen cross-link density and stiffness [258]. This transient softening of the ECM after CCRT could be exploited by consolidation immunotherapy, as a less stiff matrix suppresses PD-L1 expression [91] and restores T-cell infiltration [259]. The clinical effectiveness of this approach [260–266] suggests that using CCRT-induced ECM softening as a temporal primer for immunotherapy is biologically plausible.

Future clinical strategies should integrate biomechanical monitoring with precision and combination therapeutic approaches. While matrisome-guided targeting, mechanotransduction inhibition, and tumor-targeted delivery systems remain promising for reducing off-target effects and limiting pro-invasive remodeling [172, 179, 245, 246], increasing emphasis should be placed on real-time biomechanical assessment during treatment. Emerging modalities such as MRE and US-SWEI may enable personalized mechanical mapping of tumors by longitudinally monitoring stiffness and therapy-induced remodeling. Such approaches can identify the transient mechanical windows, thereby optimizing the timing and dosing of subsequent treatments for maximum drug penetration and therapeutic response. Furthermore, integration of patient-specific biomechanical imaging data with computational modeling and digital twin platforms may allow simulation of drug distribution, ECM remodeling, and treatment outcomes before actual administration. Combining biomechanical imaging, biomarker-guided monitoring, and adaptive dosing strategies may improve therapeutic precision while minimizing risks of invasion, resistance, fibrosis, and immune suppression [177].

Overall, the optical and US-based techniques reviewed here enable the quantitative mapping of elasticity dynamics in physiologically relevant models to bridge the gap between in vitro experimentation and clinical reality. In cancer research, spatiotemporal elasticity maps provide insights into tumor progression and mechanical responses of tumors to therapy. In tissue engineering and regenerative medicine, noninvasive elasticity mapping enables long-term monitoring of engineered constructs to provide feedback for optimizing bioreactor conditions and scaffold designs. To ensure these methods are routinely implemented, future efforts may focus on converting specialized millimeter-scale and microfluidic setups into robust protocols compatible with high-throughput screening. Achieving this alongside the development of automated analysis pipelines will transform engineering innovations into accessible platforms for investigating fibrotic diseases, wound healing, and mechanobiology-driven cancer treatments.

## Data Availability

No datasets were generated or analysed during the current study.
